# Characteristics of Foreign-Born Persons in the Swiss Hepatitis C Cohort Study: Implications for Screening Recommendations

**DOI:** 10.1371/journal.pone.0155464

**Published:** 2016-05-26

**Authors:** Barbara Bertisch, Fabio Giudici, Francesco Negro, Darius Moradpour, Beat Müllhaupt, Alberto Moriggia, Janne Estill, Olivia Keiser, Swiss Hepatitis C Cohort Study

**Affiliations:** 1 Institute of Social and Preventive Medicine, Bern, Switzerland; 2 Checkpoint Zürich, Zürich, Switzerland; 3 Divisions of Gastroenterology and Hepatology and of Clinical Pathology, University Hospitals, Geneva, Switzerland; 4 Division of Gastroenterology and Hepatology, Centre Hospitalier Universitaire Vaudois, University of Lausanne, Lausanne, Switzerland; 5 Swiss Hepato-Pancreato-Biliary Center and Department of Gastroenterology and Hepatology, University Hospital, Zürich, Switzerland; 6 Epatocentro Ticino Foundation, Lugano, Switzerland; Centers for Disease Control and Prevention, UNITED STATES

## Abstract

**Background:**

Switzerland recommends individuals who originate from high-prevalence countries to be screened for hepatitis C virus (HCV) infection. However, not all these persons are equally at risk. We thus aimed to describe the number and characteristics of persons with HCV infection born outside of Switzerland.

**Methods:**

We compared characteristics of anti-HCV-positive individuals in the Swiss Hepatitis C Cohort Study (SCCS) and of HCV cases reported to the Federal Office of Public Health (FOPH), with those of the general population in Switzerland. Persons who inject drugs (PWID) and persons who do not inject drugs (non-PWID) were compared by age groups for different countries of origin (represented by ≥1% of participants in the SCCS or FOPH).

**Results:**

We included 4,199 persons from the SCCS and 26,610 cases from the FOPH. Both groups had similar characteristics. In both data sources non-PWID were more frequent in foreign-born than in Swiss-born persons (63% versus 34% in the SCCS). The only subgroup with a clearly higher proportion both in the SCCS and FOPH than in the general population were persons over 60 years from Italy and Spain, with a 3.7- and 2.8-fold increase in the SCCS. These persons were non-PWID (99%), less frequently HIV- and anti-HBc positive and more often female than PWID from Italy and Spain; cirrhosis at enrolment was frequent (31%). Their HCV genotypes were consistent with those observed in elderly non-PWID of their birth countries. In the FOPH a higher proportion than in the general population was also seen for cases from Georgia and Russia.

**Conclusion:**

The identification of subgroups in which HCV infection is particularly frequent might allow for better targeting HCV screening among foreign-born persons in Switzerland and elsewhere.

## Introduction

The prevalence of antibodies against hepatitis C virus (HCV) in Switzerland is estimated at ≤1,8% [1–4] with the majority of HCV infections still undiagnosed [2,5–7]. Recent studies have shown that the proportion of foreign-born persons among HCV patients is high [1,5,6]. To maximize HCV detection among foreign-born persons, the Swiss Federal Office of Public Health (FOPH) and the Swiss Experts in Viral Hepatitis (SEVHep) have recommended screening for HCV in persons who were born or who have lived in countries with a HCV prevalence of ≥2% [2,6]. Prevalence is based on estimates issued by the World Health Organization (WHO) [8].

In Switzerland, 23.9% of the population are foreign-born [9], with people from Germany constituting the largest migrant group. However, for many decades since 1945, the majority of immigrants arrived from Italy, followed by the former Yugoslavia, Portugal, France, Turkey and Spain [9,10]. All these countries are in regions with an estimated HCV prevalence of ≥2% [8], but the source and frequency of HCV infection may differ between different migrant groups.

Many foreign-born persons in Switzerland come from countries where injecting drug use is the main source of HCV transmission [2,5,11–13]. Switzerland has excellent facilities for treating persons who inject drugs (PWID), and HCV screening has been recommended among PWID since 1993 [2]. Uptake of HCV screening among PWID has been shown to be high [14], and it is therefore unlikely that many foreign-born PWID remain undiagnosed.

Worldwide, most persons were infected with HCV by unsafe medical procedures [12,15]. This has also been described in some countries where foreign-born persons in Switzerland originated. For example in Italy between 1950 and 1970, laypersons injected vitamins and nutrients using reusable syringes potentially contaminated with bloodborne pathogens [16–18]. Hospitalizations, blood transfusions and parenteral tuberculosis treatments were other sources of infection [11,16,18]. In Spain, Poland and Romania, people were often infected by exposure to contaminated medical equipment [11,19] and in Bosnia, blood donor testing was not introduced until 1995 [19]. Transmission routes may have been similar in foreign-born persons, before they migrated to Switzerland. Characteristics of the affected persons and the virus genotype may reveal that the disease was transmitted to persons without (a history of) injecting drug use (non-PWID). For example in Italy, HCV infections by unsafe medical procedures mostly occurred during the period between 1950 and 1970 [11,13,16–18,20,21] and affected men and women equally [16–18,20], while HCV infections in PWID peaked between 1980 and early 2000 [16,22] and occurred mostly in men [16]. The genotype distribution and rates of coinfection with hepatitis B and HIV also differ between non-PWID and PWID in Italy [11–13,16–18,20–22]. If persons with diagnosed HCV infection in Switzerland had similar characteristics as their peers in other countries, these may help identify foreign-born persons in the general population in Switzerland at higher risk of HCV infection. Identifying the stage of liver disease by age could help us, in turn, identify people who would benefit most from treatment. We thus aimed to describe the number and characteristics of persons with HCV infection who were born outside of Switzerland.

## Methods

The two main data sources we used for this analysis were the Swiss Hepatitis C Cohort Study (SCCS) and HCV surveillance data from the Swiss FOPH. The SCCS is a multicentre cohort study with continuous enrolment of persons aged 18 years and older who are anti-HCV positive, with no exclusion criteria other than age less than 18 years [23]. Study sites include five university hospitals, three additional large hospitals, and some affiliated centres. Enrolment started in 2000, and persons in any stage of disease are included. The standardized questionnaire includes data on demographic characteristics, HCV risk factors and genotype, progression of liver disease, and concomitant infections like HBV and HIV. Information is collected at enrolment and during yearly follow-up visits. Patients have compulsory health insurance and HCV treatment is reimbursed for all patients, unless when the new direct acting antivirals are prescribed, in which case the reimbursement is guaranteed for patients who have a Metavir fibrosis stage 2 or higher [24]. The ethics committees of all eight participating hospitals granted ethical approval (Ethikkommission Nordwest- und Zentralschweiz (Basel); Kantonale Ethikkommission Bern; Commission cantonale d'éthique de Genève; Commission cantonale d'éthique de la recherche sur l'être humain du Vaud (Lausanne, Neuchâtel); Comitato etico cantonale del cantone Ticino (Lugano); Ethikkommission des Kantons St. Gallen; Kantonale Ethikkommission Zürich). All participants gave written informed consent.

In 1988, the FOPH started including HCV infections in its mandatory national surveillance system. All persons who are anti-HCV positive, and whose infection is confirmed by immunoblot or PCR, must be reported within a week of diagnosis both by the treating physician and the laboratory [25]. The standardized forms are sent to the Cantonal Physician; the laboratory form is also sent to the FOPH. The form for physicians [25] includes demographic characteristics like nationality and country of origin, presumed route of exposure to HCV and presence of cirrhosis or hepatocellular carcinoma at diagnosis. The laboratory form contains the date of blood sampling, test date and test method. Both forms contain the name of the physician and the laboratory [25]. A reminder is sent in case either report is missing. The estimated completeness of reporting is high, and notification forms are checked for double reporting. Participants of the SCCS are also included in the FOPH registry, but a direct linkage is not possible. In either registry, some persons had been diagnosed with HCV infection following presentation of signs of liver disease/complications or in the presence of risk factors; others had undergone screening for other reasons (e.g. blood donation). While injecting drug use had long been known to be a risk factor, many sites screened persons with other risk factors including persons from high-prevalence countries long before this had become an official recommendation in 2013/14 [2,6].

The Swiss Federal Statistical Office (FSO) provided data on the number of foreign-born persons by age group in the general Swiss population. Since data were only available for 1990, 2000 and 2010–2013, we used the data from 2010–2013 in the main analysis, but conducted a sensitivity analysis using data from 1990 and 2000.

### Inclusion criteria and definitions

We included all persons with a HCV infection enrolled in the SCCS (up to April 2014) in the analysis, and all cases included in the FOPH registry, aged 18 or older (up to July 2014). Persons with/without a history of past or ongoing injecting drug use were classified as PWID/non-PWID. We wanted to reflect the age distribution of people who would need to be screened in a realistic way; thus we calculated the age of all persons as of 1^st^ of January 2014. We used the United Nations geographical region classification to group persons with a HCV infection by country and region of birth [26]. Since country of birth is not available in the FOPH, we used country of origin (the country from which the case or the case’s parents had migrated). If this was unavailable, we used nationality as a proxy. Bosnia and Herzegovina, Kosovo, Croatia, Macedonia, Montenegro, Serbia and Slovenia were all categorized as “former Yugoslavia”.

### Data analysis

We conducted four descriptive analyses: First, we grouped both Swiss and foreign-born persons/cases by age and presence/absence of a history of injecting drug use. We did this for different regions (Northern, Southern, Eastern and Western Europe; Africa; Asia/Oceania; America) or by country of birth or origin (represented by ≥1% of persons in the SCCS/FOPH). We displayed these results graphically. Second, within each country, we calculated the prevalence ratio in the SCCS/FOPH to the general population by 5-year age groups. We used the test for the equality of proportions (based on the z test) to calculate overrepresentation. Third, differences between PWID and non-PWID were tested using χ2 tests or Fisher's exact tests for categorical variables (i.e. gender, HIV/HBV status and genotype), and the test for equality of medians for continuous variables (i.e. age).

Lastly, we compared the prevalence and incidence of cirrhosis among the different age groups and countries of birth in the SCCS.

We then evaluated modifiable lifestyle factors that influence the progression of liver disease [27,28] (i.e. alcohol consumption (“person ever used to drink >20g alcohol/day” [yes/no]), body mass index [BMI] and diabetes mellitus). From the FOPH, no comparable data were available.

## Results

We included in the analysis a total of 4,199 persons in the SCCS, representing 99% of all persons enrolled (n = 4,252) whose age and country of birth were known, and whose information on presence/absence of past or ongoing injecting drug use was available. The characteristics of the SCCS study participants are shown in Table 1 and in S1 Table; 32% were foreign-born (thus similar to 30% of foreign-borns in the general adult population); 63% were male and the median age was 51 years. From the FOPH registry, age, country of origin and presence/absence of past or ongoing injecting drug use were only available for 56% of cases (26,610/47,754). S2 Table shows all FOPH registry data, S3 Table has the characteristics of included and excluded cases, and S4 Table contains all data of the included cases. Cases were mostly excluded due to missing information on drug use (83% missing) and country of origin (56% missing) while age was missing in less than 1% of all cases. Characteristics of persons/cases were similar between included and excluded persons in the FOPH and between the SCCS and FOPH groups. The main difference between the SCCS and FOPH groups was in the time from diagnosis to enrolment as these two dates coincide in the FOPH due to the mandatory reporting.

**Table 1:** Characteristics of persons with a HCV infection in the Swiss Hepatitis C Cohort Study (SCCS) and of cases mandatorily reported to the Federal Office of Public Health (FOPH).

HCV registry	SCCS (2000–2014)			FOPH (1988–2014)		
	*Swiss-born*	*Foreign-born*	*All*	*Swiss-born*	*Foreign-born*	*All*
**Total number**	2,863*	1,357*	4,252	24,880*	11,032*	47,754
**Number analysed†**	2,848	1,351	4,199 (99% of total number)	18,655	7,955	26,610 (56% of total number)
**Age (years) at HCV diagnosis (median, IQR)**	35 (27–43)	39 (31–50)	36 (28–45)	36 (28–45)	37 (29–48)	36 (29–46)
**Age (years) as of Jan 2014 (median, IQR)**	50 (44–57)	53 (45–62)	51 (45–59)	50 (44–57)	48 (40–58)	49 (43–57)
**From HCV diagnosis to enrolment (years)**	5 (1–10)	4 (1–9)	5 (1–10)	0	0	0
**Male gender**	1,826 (64%)	812 (60%)	2,638 (63%)	11,291 (61%)	5,218 (66%)	16,509 (62%)
**Non-PWID**	956 (34%)	855 (63%)	1,811 (43%)	6,369 (34%)	4,414 (55%)	10,783 (41%)
**Country of birth/origin, by order of frequency**						
	Italy 395 (9%)			Italy 2,224 (8%)		
	Germany 104 (2%)			Portugal 650 (2%)		
	Former Yugoslavia 85 (2%)			Former Yugoslavia 497 (2%)		
	Portugal 83 (2%)			Spain 473 (2%)		
	France 81 (2%)			Georgia 460 (2%)		
	Spain 72 (2%)			Russia 323 (1%)		
	Austria 54 (1%)			Germany 298 (1%)		
	Others 477 (11%)			France 282 (1%)		
				Others 2,748 (10%)		

† = only persons/cases were included where age, presence/absence of past or ongoing injecting drug use and country of birth (SCCS)/of origin (FOPH) were known.

* = Numbers reduced due to missing information. Percent values refer to the total number analyzed, unless otherwise stated. Non-PWID = persons who do not inject drugs.

**Fig 1:**
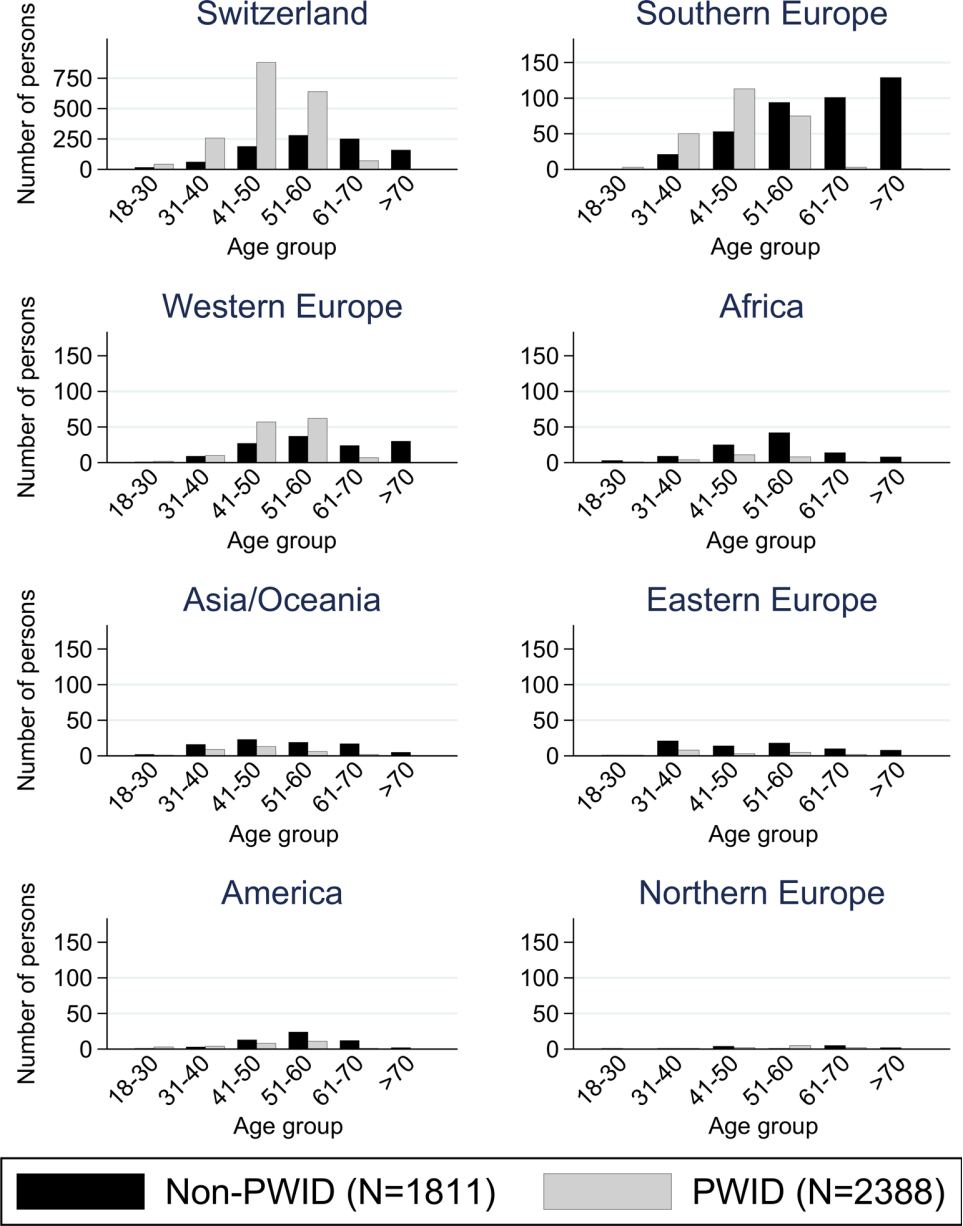
Persons who do not inject drugs (non-PWID) versus persons who inject drugs (PWID) in the Swiss Hepatitis C Cohort Study by region of birth and age (please note the different y-axis scale for Switzerland).

In the SCCS, 68% (2,848/4,199) of the persons analyzed were Swiss-born. In Swiss-born persons the majority were PWID (Table 1). The age distribution was similar between PWID and non-PWID (Fig 1). In contrast, in foreign-born persons the majority were non-PWID (Table 1) and age clearly differed between PWID and non-PWID (Fig 1). Most non-PWID originated from Southern Europe (Fig 1) and in particular from Italy and Spain (S5 Table).

Regions of birth are classified according to the United Nations geographical region classification and displayed by frequency. Age is in years as of January 2014. PWID = persons who inject drugs; non-PWID = persons who do not inject drugs.

**Fig 2:**
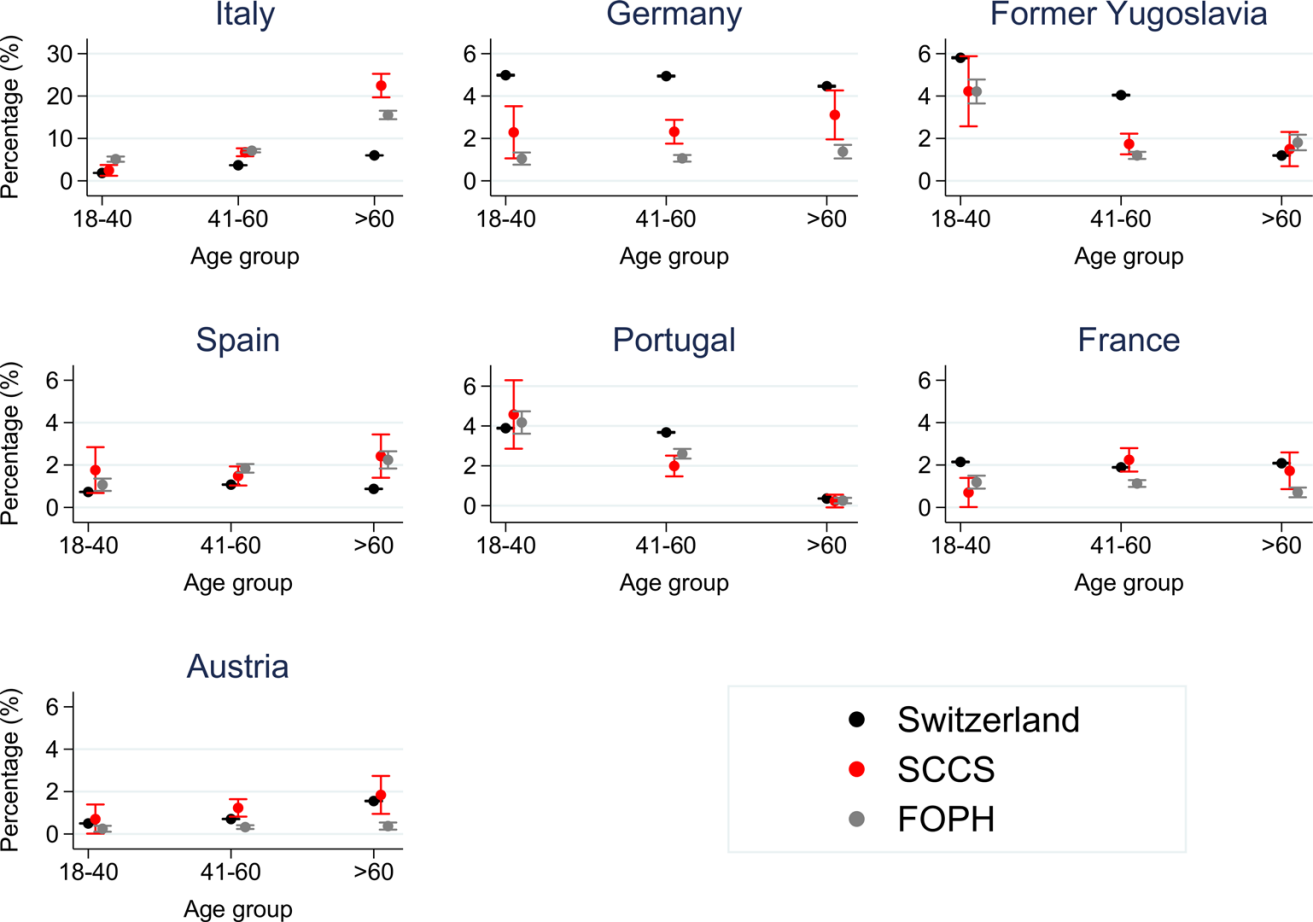
Prevalence of country representatives by age group in the Swiss Hepatitis C Cohort Study (SCCS), in cases mandatorily reported to the Federal Office of Public Health (FOPH) and in the general population of Switzerland (please note the different y-axis scale for Italy).

Over a third (35%; 303/855) of all foreign-born non-PWID in the SCCS came from Italy or Spain; among these, 70% (213/303) were over 60 years. In addition, we found three PWID over 60 years from Italy and Spain. Persons over 60 years from Italy and Spain showed a clear increase in their proportion both in the SCCS and FOPH compared to their proportion in the general population (Fig 2; S1, S2 and S4 Tables), with a 3.7-fold increase for Italian-borns and a 2.8-fold increase for Spanish-borns in the SCCS (combined data from Table 2). Some other groups from Italy and Spain (i.e. Italian-born persons aged 41–55 and Spanish-born persons aged 36–40 and 46–50) were also overrepresented in both the SCCS and the FOPH. However, the overrepresentation was moderate (Table 2) and mostly caused by PWID (S5 Table). No other migrant/age group was overrepresented in both data sources (Table 2).

**Table 2:** Ratio of proportions in the Swiss Hepatitis C Cohort Study (SCCS) respectively in cases mandatorily reported to the Federal Office of Public Health (FOPH) compared to the proportion in the general population, by country of birth/origin and age group.

**Age group**	**ITALY**	**GERMANY**	**FRANCE**	**SPAIN**	**PORTUGAL**	**AUSTRIA**	**YUGOSL.**
**18–25**	0.0/2.2	3.5/0.2	0.0/0.7	0.0/1.4	3.4/1.0	0.0/0.0	0.0/0.5
**26–30**	0.0/1.4	0.5/0.3	0.0/0.5	0.0/0.8	0.0/0.5	0.0/0.8	0.4/0.5
**31–35**	0.6/**1.9**	0.4/0.2	0.5/0.4	1.9/0.6	0.8/0.8	**2.9**/0.1	0.9/0.8
**36–40**	1.4/**2.5**	0.3/0.2	0.2/0.5	**2.1/1.5**	1.3/1.1	0.5/0.5	0.8/0.8
**41–45**	**1.8/3.1**	0.3/0.2	0.8/0.4	1.4/**2.4**	0.4/0.9	1.7/0.7	0.3/0.4
**46–50**	**2.1/2.5**	0.4/0.2	1.2/0.8	**2.2/2.0**	0.4/0.6	1.5/0.4	0.5/0.2
**51–55**	**1.6/1.3**	0.6/0.2	1.1/0.7	1.4/**1.5**	0.6/0.6	1.2/0.4	0.4/0.2
**56–60**	**1.7/**1.1	0.8/0.5	**1.8**/0.5	0.4/1.1	0.9/0.7	**3.0**/0.3	0.5/0.5
**61–65**	**2.2/1.4**	1.1/0.3	1.1/0.4	1.7/1.2	0.4/0.6	2.0/0.5	0.8/1.2
**66–70**	**3.7/2.7**	0.5/0.2	0.7/0.4	2.0/**3.1**	0.0/0.8	0.4/0.0	1.9/**2.7**
**71–75**	**4.8/3.4**	0.5/0.5	0.6/0.4	**3.5/2.8**	0.0/1.7	1.0/0.2	1.3/1.7
**76–80**	**5.0/3.5**	0.4/0.2	0.4/0.4	**3.8/3.9**	0.0/1.0	1.5/0.3	0.0/1.6
**81–85**	2.2/**3.4**	1.4/0.1	1.2/0.2	**9.0/4.1**	**34.1**/0.0	2.6/0.2	0.0/**2.8**
**>85**	2.1/**2.2**	**5.4**/0.4	3.8/0.2	0.0/**4.4**	0.0/1.9	0.0/0.4	0.0/2.0

The countries of birth/of origin of ≥1% of the persons in the SCCS/ cases in the FOPH are shown. Age is in years as of January 2014. The bars show the 95% confidence intervals.

The countries of birth/of origin of ≥1% of the persons in the SCCS/cases in the FOPH are shown. Age is in years as of January 2014. The first/second number is the ratio with data from the SCCS/FOPH. Bold numbers = ratio significantly increased. In Italian-borns aged 61–80 and Spanish-borns aged 71–85, the ratio is significantly increased both in the SCCS and FOPH, almost exclusively (99%) in non-PWID. In Italian-borns aged 41–55 and Spanish-borns aged 36–40 and 46–50, the ratio is significantly increased both in the SCCS and FOPH, by a majority in PWID. Yugosl. = former Yugoslavia

The characteristics of non-PWID born in Italy and Spain clearly differed from PWID: non-PWID had a more balanced gender distribution, a lower rate of HBV and HIV coinfection, and different genotypes with more frequent genotype 1b and 2. The differences in person characteristics and genotypes were more pronounced in non-PWID over 60 years (Table 3).

**Table 3:** Italian- and Spanish-born persons in the Swiss Hepatitis C Cohort Study: comparison of persons who do not inject drugs (non-PWID) ≤60 years and >60 years versus persons who inject drugs (PWID) of all ages.

		Non-PWID ≤60 years (n = 90)	Non-PWID >60 years (n = 213)	PWID* (n = 164)	P-value non-PWID ≤60 years vs PWID
**Age as of 2014**	**Median (IQR)**	53 (48–56)	72 (67–75)	48 (44–53)	p = 0.007
	**Min-max**	32–60	61–87	33–73	
**Male gender**		59 (66%)	119 (56%)	133 (81%)	p = 0.006
**HIV status**	**Available**	64 (71%)	134 (63%)	143 (87%)	
	**HIV positive (% of available)**	3 (5%)	1 (1%)	21 (15%)	p = 0.058
**Anti-HBc serology**	**Available**	84 (93%)	194 (91%)	154 (94%)	
	**Anti-HBc positive (% of available)**	32 (38%)	66 (34%)	78 (51%)	p = 0.063
**Genotype**	**Available (% of (non-) PWID**	89 (99%)	201 (94%)	148 (90%)	
	**1, subtype unknown**	20 (22%†)	67 (33%†)	35 (24%†)	p = 0.835
	**1a**	8 (9%†)	6 (3%†)	16 (11%†)	p = 0.653
	**1b**	24 (27%†)	59 (29%†)	14 (9%†)	p<0.001
	**2**	11 (12%†)	58 (29%†)	4 (3%†)	p = 0.005
	**3**	19 (21%†)	4 (2%†)	60 (40%†)	p = 0.002
	**4**	7 (8%†)	7 (3%†)	19 (13%†)	p = 0.236

*with only 3 persons aged >60 years

† % of available genotypes

The state of cirrhosis in the SCCS at enrolment and during follow-up can be seen in Table 4.

**Table 4:** Cirrhosis at enrolment and during follow-up in different age groups and countries of birth in the Swiss Hepatitis C Cohort Study.

Age	Cirrhosis at enrolment*		Child-Pugh score A*		Cirrhosis during follow-up†¥		Child-Pugh score A†	
	*Italy*, *Spain*	*Other countries*	*Italy*, *Spain*	*Other countries*	*Italy*, *Spain*	*Other countries*	*Italy*, *Spain*	*Other countries*
**18–50**	8% (11/138)	6% (121/1,865)	73% (8/11)	79% (96/121)	6% (8/127; 1.8)	6% (109/1,744; 1.8)	100% (8/8)	90% (98/109)
**51–60**	32% (36/113)	18% (217/1,215)	69% (25/36)	79% (172/217)	14% (11/77; 3.2)	11% (115/998; 2.7)	73% (8/11)	94% (108/115)
**61–70**	26% (24/91)	27% (117/432)	96% (23/24)	83% (97/117)	19% (13/67; 3.8)	17% (53/315; 3.6)	92% (12/13)	87% (46/53)
**71–80**	33% (39/118)	28% (52/183)	85% (33/39)	85% (44/52)	20% (16/79; 3.8)	18% (23/131; 3.7)	94% (15/16)	96% (22/23)
**>80**	43% (3/7)	27% (10/37)	100% (3/3)	80% (8/10)	25% (1/4; 5.8)	11% (3/27; 2.9)	100% (1/1)	100% (3/3)
**All**	15% (630/4,199)		81% (509/630)		10% (352/3,569; 2.5)		92% (321/352)	

Age is as of January 2014, in years.

* = of persons in age group

† = of remaining persons in age group

¥ = with incidence rate per 100 person-years

Generally, the rate of cirrhosis increased with age. Persons from Italy/Spain had similar rates of cirrhosis at enrolment and during follow-up as people from other countries (with the exception of people aged 51–60 years where cirrhosis at enrolment was higher in people from Italy/Spain compared to other countries). Most patients with cirrhosis were still in Child-Pugh class A. Among Italian- and Spanish-born persons over 60 years, 31% (66/216) had cirrhosis at enrolment into the SCCS; during follow-up, another 20% (30/150) developed cirrhosis.

At enrolment in the SCCS, 22% (47/213) of Italian- and Spanish-born non-PWID over 60 years had a history of increased alcohol use, 16% (35/213) had a BMI >30 kg/m^2^, and 12% (26/213) had been diagnosed with diabetes mellitus (S1 Table).

**Fig 3:**
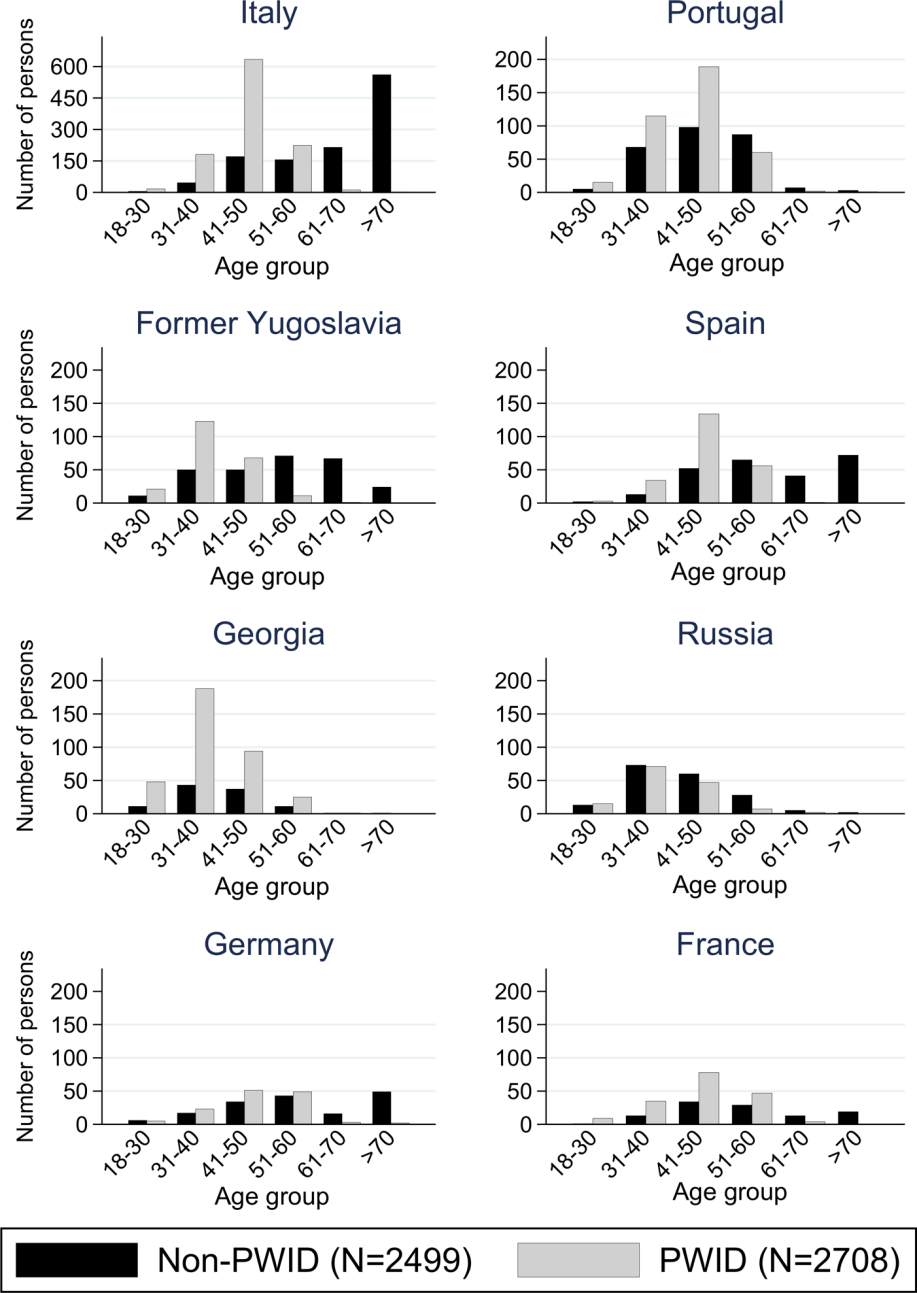
Persons who do not inject drugs (non-PWID) versus persons who inject drugs (PWID) among foreign-born persons in the national surveillance data of the Federal Office of Public Health, by country of origin and age (please note the different y-axis scale for Italy).

In the FOPH, in addition a significantly higher percentage of cases originated from Georgia (mostly PWID) and from Russia (PWID and non-PWID, Fig 3) than what is found in the general population in Switzerland.

The countries of origin of ≥1% of the cases in the FOPH are shown. Country of origin is defined as the country from which the case or the case`s parents had migrated; if unavailable, nationality was taken as a proxy. Age is in years as of January 2014. PWID = persons who inject drugs; non-PWID = persons who do not inject drugs.

## Discussion

Our study shows that among persons with already diagnosed HCV infection in Switzerland, the rate of foreign-born persons is similar to their rate among the general population in Switzerland. Among foreign-born persons with HCV infection, non-PWID are much more frequent than among Swiss-born persons (63% versus 34% in the SCCS). However, only the proportions of persons born in Italy and Spain over 60 years (and therefore born before 1953) are clearly overrepresented (by >2.8 fold) compared to the general population in Switzerland. The higher prevalence of HCV and the characteristics of these older persons from Italy and Spain reflect the situations in their birth countries, where healthcare-associated infections were frequent between 1950 and 1970 [11–13,16–18,20,21,29–31]. The characteristics of HCV-infected non-PWID differ from those of HCV-infected PWID born in the same countries, with the most pronounced difference for persons born before 1953. Non-PWID born before 1953 are more likely to be women, are less likely to have serological markers of HIV- and hepatitis B virus infection, and are infected predominantly by HCV genotype 1b and 2 [17,18,20,29,30]. Thus, the healthcare-associated HCV infections in persons over 60 years that are reported from Italy are also present among Italian-born persons over 60 years in Switzerland.

Although our findings apply to Switzerland and reflect a predominantly European migration, they may also apply to other countries. In Belgium, for example, older non-PWID from Italy were common among HCV infected persons [32]. Germany was the main destination for Italian migrants from 1961 to 1980 and accommodated over 600,000 of them [33]. Two German studies detected a high HCV prevalence in persons from Eastern Europe and Russia, but found no association for persons from Italy [34,35]. However, the inclusion criteria of these studies may explain their results. Migration was defined by mother tongue [34] and studies included second-generation migrants born in Germany, who had a low risk of HCV infection [35]. Italian immigrants may also have been underrepresented in these studies since they are not homogenously distributed in Germany [36].

France and Germany were the main destinations for those who migrated from Spain between 1960 and 1973 [37]. However, we found no studies from France on the HCV prevalence in Spanish-born persons, and the inclusion criteria of the above-mentioned two German studies may explain why no higher HCV prevalence was seen for persons from Spain [34,35].

In the Netherlands and the UK, many patients with chronic hepatitis C come from highly endemic non-European regions like Northern Africa (particularly Egypt [38]) and the Indian subcontinent (particularly Pakistan), but far fewer of these immigrants are living in Switzerland.

In the UK, testing migrants for HCV infection was deemed to be cost-effective [39]. In the Netherlands, a pilot campaign on HCV screening was aimed at the general public, with a typical representation of both migrants and PWID; this program was also considered cost-effective [40]. We are not aware of any cost-effectiveness analysis of screening older migrants. Although we did not perform a cost-effectiveness analysis, we believe that advanced age and the potential benefit of therapy after screening should be taken into account when considering the cost of HCV screening. In our study, we found a high rate of cirrhosis, with increasing prevalence and incidence as a function of age. Based on their high rates of cirrhosis at enrolment and during follow-up, a significant number of non-PWID from Italy and Spain could benefit from therapy. New treatments for chronic hepatitis C (although expensive) may clear HCV in the vast majority of patients [41,42]; liver disease progression with costly interventions could be avoided. A considerable number of non-PWID from Italy and Spain have been diagnosed with alcohol consumption >20g, obesity and diabetes mellitus at or before enrolment. Knowledge of the HCV infection may contribute to intensify interventions to manage these additional factors of accelerated liver disease progression [27,28].

Our study has both strengths and limitations. It was strengthened by use of data from two complementary sources. The FOPH registry includes ten times more cases than there are persons in the SCCS, and the cases are in a less advanced stage of the disease. The FOPH data therefore reflects the HCV-positive population in Switzerland and the screening situation more closely. Because of missing values in the FOPH data, we had to exclude 44% of cases from the analysis. It is unknown how this affects the results, however the characteristics of included and excluded cases were similar. Thus, we do not expect a relevant change of the results to be caused by the missing values. The FOPH data also has only a limited number of variables and a cross-sectional design. These data overestimate the number of foreign-born persons, as persons born in Switzerland after their parents had migrated are miss-categorized as foreign-born. However, this is unlikely to have much impact on Italian- and Spanish-born persons over 60 years, since mass migration to Switzerland from Italy and Spain started after 1950 [33,37]. The FOPH database covers a longer time-span than the SCCS database, but we obtained similar results when we restricted the FOPH analysis to the same time period as the SCCS. When we used FSO data from 1990 and 2000 instead of 2010–2013, results were similar (not shown). The study has a number of additional limitations: First, data from the SCCS and the FOPH are based on diagnosed cases, while the majority of HCV infections in Switzerland are undiagnosed. Second, non-PWID may be less frequently diagnosed and therefore underrepresented because screening traditionally focused on PWID. Similarly, older people may have been diagnosed more frequently than younger ones because liver complications increase with age. Third, since the SCCS aims to follow persons over an extended time period, persons with an anticipated limited duration of their stay in Switzerland might have been excluded (e.g. asylum seekers and persons who come to Switzerland to be treated). This may explain why more Georgian- and Russian-born cases were recorded in the FOPH (but not in the SCCS) than in the general population in Switzerland. Fourth, some groups with known high HCV prevalence (e.g. people from Egypt) were infrequent, as their number in Switzerland is very low. Fifth, access to healthcare may vary between Swiss- and foreign-born people from different countries and between age groups within countries of origin. Sixth, in non-PWID, we can only speculate that transmission occurred mostly via unsafe medical procedures, as this is the most likely explanation.

In conclusion, although our data are based on diagnosed cases only and therefore need to be confirmed, our findings could be used to supplement the HCV screening recommendation issued by the FOPH and SEVHep which is a provider-initiated counseling and testing (PICT) strategy with focus on general practitioners (GPs) [2,6]. GPs are used to PICT strategies: PICT is also used for HIV screening [43], and the HIV and HCV PICT strategies have been repeatedly published for GPs in Switzerland [2,6,43,44]. GPs have good access to older people, as older people attend GPs more frequently than the general population in Switzerland [45]. The implementation of HCV screening with a focus on Italian- and Spanish-born persons over 60 years should therefore be feasible. Our study would thus help to identify, among the Swiss general population, foreign-born persons at increased risk of HCV infection.

## Supporting Information

S1 Table Data from the Swiss Hepatitis C Cohort Study used for this study. (XLSX)

S2 Table Data from the hepatitis C registry at the Federal Office of Public Health used for this study. (XLSX)

S3 Table Characteristics of included and excluded cases in the hepatitis C registry at the Federal Office of Public Health. (XLSX)

S4 Table Data from the Swiss Federal Statistical Office used for this study. (XLSX)

S5 Table SCCS and FOPH: all persons/cases by age group and country of birth/origin. (XLSX)
